# Biomechanical analysis of trunk and lower limbs during stair activity in patients with scoliosis

**DOI:** 10.1038/s41598-024-65665-2

**Published:** 2024-06-24

**Authors:** Yanyun Gou, Jing Tao, Jia Huang, Huangwei Lei, Xiang Chen, Xiangbin Wang

**Affiliations:** Fujian University of Traditional Chinese Medicine, No.1 Qiuyang Road, Minhou Shangjie, Fuzhou, 350122 Fujian China

**Keywords:** Scoliosis, Motion analysis, Ascending and descending stairs, Kinematics and kinetics, Health care, Signs and symptoms

## Abstract

Staircases are a frequently encountered obstacle in daily life, requiring individuals to navigate ascending and descending movements that place additional demands on the trunk and lower limbs compared to walking on level surfaces. Therefore, it is crucial to examine the biomechanical characteristics of the trunk and lower limbs in individuals with scoliosis during stair activity. The aim of this study was to investigate the biomechanical differences in trunk and lower limbs during daily stair activities between patients with scoliosis and a healthy population. Additionally, the study aimed to explore the relationship between trunk abnormalities and lower limb biomechanics, providing a clinical and objective assessment basis for scoliosis. The Qualisys system, based in Gothenburg, Sweden, was employed for data collection in this study, with a sampling frequency of 150 Hz. It captured the kinematics of the trunk and lower limbs, as well as the kinetics of the lower limbs during stair ascent and descent for both the 28 individuals with scoliosis and the 28 control participants. The results indicate that scoliosis patients demonstrated significantly higher asymmetry compared to the control group in various measures during ascent and decent. These include different parts of kinematics and kinetics. Scoliosis patients demonstrate noticeable variations in their movement patterns compared to the healthy population when engaging in stair activities. Specifically, during stair ascent, scoliosis patients exhibit a seemingly more rigid movement pattern, whereas descent is characterized by an unstable pattern.

## Introduction

Stairs are obstacles encountered frequently in daily life. Going up and down stairs is a more challenging task than walking on a flat surface, requiring higher demands on the trunk and lower limbs^1^, while also increasing the risk of falling^2^. Negotiating stair movement relies on the coordinated rhythm and muscular adaptation between the trunk and lower limbs, which are crucial for maintaining body equilibrium and stability^3^. Any impairment to the muscular, skeletal, or nervous systems can result in deviations during stair-related activities. Various diseases may be compensated for through adjustments in movements across different regions. Notably, scoliosis represents the most prevalent spinal disorder that significantly impacts the efficiency of locomotion^4^.

The process of ascending and descending stairs demonstrates distinct biomechanical characteristics in the trunk, pelvis, and lower extremities. Presently, the analysis of functional movement in relation to scoliosis is primarily limited to analyzing gait on level surfaces. Nevertheless, multiple studies have revealed that negotiating stairs poses greater challenges and necessitates enhanced trunk control^5–7^. Research affirms that this activity demands heightened coordination and core stability in order to maintain equilibrium and control^8,9^. This proves particularly challenging for individuals with scoliosis, as they may encounter limitations in their ability to control their trunk^10,11^. Assessing the performance of these patients during stair-related activities provides a more comprehensive comprehension of their movement abilities and functional ability.

Multiple studies on scoliosis primarily focus on the biomechanics of the trunk or lower limbs^4,12,13^, while fewer analyses investigate the relationship between them. Several studies have demonstrated that spinal diseases can result in joint or muscle damage in the lower limbs, leading to various issues, including pain and arthritis^14,15^. This correlation may be attributed to the three-dimensional deformity of the scoliosis, which causes abnormal spinal movement in the horizontal plane, consequently affecting the joint load and muscle contraction in both lower limbs. As a result, studying the biomechanics of the spine and lower limbs in patients with scoliosis can significantly contribute to the prevention of possible lower limb functional impairments in the future.

This study investigates alterations in spinal and lower limb movement patterns, as well as locomotion symmetry, during stair ascending and descending tasks among individuals with scoliosis compared to a control group through a case–control design. The study aims to summarize the abnormal changes in the spine and lower limbs that occur after the onset of scoliosis and provide an objective basis for the subsequent clinical evaluation of scoliosis.

## Materials and methods

This is a case–control study.

### Ethics statement

This study has obtained approval from the Ethics Committee of the Affiliated Rehabilitation Hospital of Fujian University of Traditional Chinese Medicine (Approval No: 2020KY-015-02). The study has been registered with the Chinese Clinical Trial Registry (registration number ChiCTR2000034580) on 10/07/2020.

### Participants

The study participants were recruited from the University Town located in Fuzhou, Fujian Province, China. Scoliosis patients were matched in a 1:1 ratio with healthy individuals (controls) according to gender.

#### Inclusion criteria for controls

(1) Participants had to be clinically diagnosed by a physician and undergo X-ray imaging (full-spine anteroposterior and lateral view) to confirm the absence of scoliosis (Cobb angle ≤ 10°). (2) They should have no history of musculoskeletal system diseases or injuries within the past 6 months. (3) Age range of 18–20 years, irrespective of gender. (4) Ability to independently ascend/descend a set of 8 stairs.

#### The inclusion criteria for scoliosis

(1) participants must meet the diagnostic criteria^16^ for scoliosis; (2) between the ages of 18 and 20, regardless of gender; (3) X-ray screening that shows a Cobb angle between 10 and 30° (excluding 10° and 30°); (4) participants must have signed an informed consent form and voluntarily agreed to participate in the trial.

#### Exclusion criteria for scoliosis

(1) presence of severe spinal deformities such as congenital, traumatic types or skin diseases in the spinal area; (2) true leg length discrepancy exceeding 2 cm on both sides; (3) BMI higher than 25.

## Assessment

### Leg length discrepancy refers to the measurement of leg length in both standing and supine positions

The functional leg length discrepancy is the distance from the belly button to the medial malleolus, while the true leg length discrepancy is measured from the anterior superior iliac spine to the medial malleolus.

### Kinematics involves the range of joint movements

This includes measuring the range of motion of the hip, knee, and ankle joints in the sagittal plane, as well as the range of motion of the hip joint in the coronal plane. The asymmetry index (AI)^17^ is used to assess the asymmetry in the movements of the hip, knee, and ankle. A higher AI number indicates a greater degree of bilateral asymmetry. In the control group^18^, the formula used is (right − left)/0.5 × (right + left). For individuals with scoliosis, the formula is (convex − concave)/0.5 × (convex + concave).

**Figure 1:**
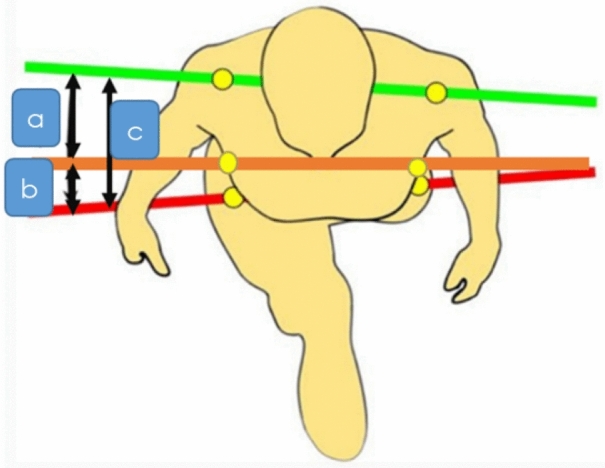
Spinal horizontal plane relative motion measurement. Image referred to [Pesenti et al.^13^.]

In the study of spinal horizontal plane relative motion^13^ represent the spinal kinematics, three relative ranges of motion were examined. The first relative range of motion involved the movement between the scapulae (bilateral acromion) and the thorax (bilateral inferior border of the 12th rib). The second relative range of motion focused on the movement between the thorax (bilateral inferior border of the 12th rib) and the pelvis (bilateral anterior superior iliac spine). Lastly, the third relative range of motion assessed the movement between the scapulae (bilateral acromion) and the pelvis (bilateral anterior superior iliac spine). These measurements can be visualized in Fig. 1.

### Kinetics

This section examines the peak moment of the hip, knee, and ankle joints in the sagittal plane. It also analyzes the antero-posterior (AP), medio-lateral (ML), and vertical (V) ground reaction forces in the same plane. Additionally, it investigates the displacement of the center of mass (COM) of the body during stair activities in the sagittal plane.

## Data collection

### Preparations prior to data collection

In line with the specifications of the three-dimensional motion analysis system (Eagle-4) developed by the Motion Analysis Corporation in the United States, markers are affixed to various anatomical sites on the subject's body. For the trunk, the markers are positioned at the 12th rib (BH) and the acromion (SH). The lower body marker model, based on CAST/Cleveland Clinic's lower limb model^19^, markers placed on the pelvis (anterior superior iliac spine, posterior superior iliac spine, highest point of the iliac crest), thigh (greater trochanter, mid-shaft of the femur, medial and lateral condyles of the femur), lower leg (mid-shaft of the tibia, medial malleolus, lateral malleolus), and foot (dorsal edge of the 5th metatarsal head, dorsal edge of the 2nd metatarsal head, dorsal edge of the 1st metatarsal head, calcaneus). Following the calibration of the motion analysis system workstation's eight cameras (sampling frequency: 100 Hz), data collection is initiated. Subsequently, the gathered data is subjected to bandpass filtering, followed by the computation and application of the correlation coefficient for further analysis.

**Figure 2:**
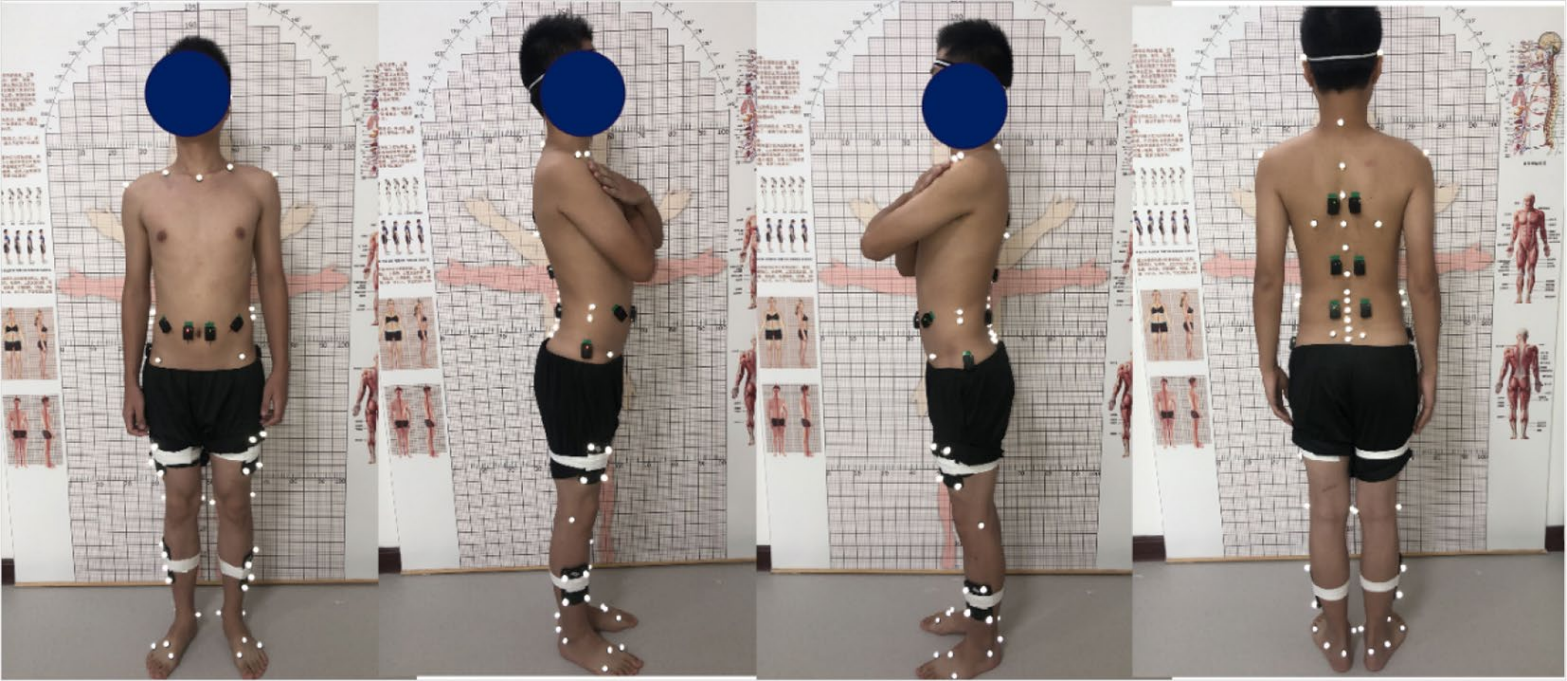
Positions of the tracking markers.

The positions of the tracking markers are displayed in Fig. 2^19,20^.

### Data collection process

Prior to data collection, participants were given a thorough introduction to the experimental protocol. To familiarize themselves with the environment and attain a comfortable walking speed, they performed a minimum of three trials of ascending and descending stairs. The participants engaged in stair ascent and descent activities on an 8-step custom adjustable staircase, with each step measuring 20 cm in height and 30 cm in depth. For measurement purposes, force plates (Kistler, 9260AA, Switzerland) measuring 60 cm × 50 cm were positioned on the third and fourth steps. Data was sampled at a frequency of 1000 Hz. In terms of motion capture data analysis, a 3D motion capture system (Qualisys, Gothenburg, Sweden) was utilized to synchronously capture both kinematic and kinetic gait data at a sampling frequency of 100 Hz.

## Data analysis

The V3D software (Visual 3D Professional V6, C-motion InC, USA) was utilized for data analysis. Marker points were named for each data collected in the static condition, and the joint coordinate systems were defined in accordance with the guidelines recommended by the International Society of Biomechanics^21^. The V3D software's model-based calculation function was employed to determine the joint angles. The inertia parameters of each segment, such as the joint center and segment mass, were calculated. A three-dimensional coordinate system was established for each segment, and the angles of each segment around each coordinate axis were obtained using matrix transformation. Subsequently, the Euler angles of segment rotation were obtained through inverse kinematics, and inverse dynamics calculations were performed based on the ground reaction force to determine joint moment and other relevant indices. The ground reaction force was standardized with respect to the subject's body weight. Furthermore, all joint moment were analyzed by standardizing them based on both body weight and height (%BM × Height).

## Statistical analysis

The original data was entered into EXCEL 2013 and data analysis was conducted using SPSS 24.0 software. For continuous data that follows a normal distribution, it is expressed as $\overline{x}$ ± *s*. Between-group comparisons are conducted using independent samples t-test. The significance level for hypothesis testing was set at *P* < 0.05. Non-parametric tests were used for data that did not follow a normal distribution.

All the methods employed in this study were conducted in strict adherence to the pertinent guidelines and regulations.

## Bias

Several measures were taken to address potential sources of bias in this study.

Efforts were made to minimize confounding variables through careful study design and statistical analysis. Covariates such as age, gender, and comorbidities were controlled for in the analysis, allowing for a more accurate assessment of the relationship between the independent and dependent variables. To ensure the reliability of data, standardized assessment tools and protocols were used, and inter-rater reliability was assessed. This minimized measurement bias and enhanced the internal validity of the study. Ethical considerations were also addressed to mitigate potential bias. Informed consent was obtained from all participants, and the study protocol was reviewed and approved by the institutional review board.

These efforts to address potential sources of bias strengthen the validity and reliability of the study findings, enhancing the confidence in the results and their interpretation.

## Results

### Participants baseline

The study included a total of 28 scoliosis patients and 28 healthy controls. There were no statistically significant differences observed between the two groups in terms of gender (scoliosis: 15 females, 13 males; control: 15 females, 13 males), age (scoliosis: 19.5 years; control: 19.5 years), height (scoliosis: 1.70 m; control: 1.64 m), weight (scoliosis: 59.25 kg; control: 60.13 kg), and BMI (scoliosis: 20.73; control: 21.28) (*P* > 0.05). The Cobb angle (scoliosis: 16.00°) was significantly higher compared to the control group (control: 5.00°) (*P* < 0.05).

### Leg length discrepancy

The results of Table 1 showed there was no statistically significant difference in the leg length discrepancy between the two groups (*P* > 0.05).

**Table 1:** Leg length discrepancy between group analysis [$\overline{x}$ ± *s*, M(P25, P75)].

Bilateral Difference (cm)	Testing Position	Scoliosis (n = 28)	Control (n = 28)	t/Z	*P* value
True leg length discrepancy	Supine^#^	0.20 (0.10,0.40)	0.20 (0.10,0.30)	− 0.217	0.828
	Standing^#^	0.10 (0.00,.030)	0.10 (0.00,0.40)	− 0/540	0.589
Functional leg length discrepancy	Supine^#^	0.10 (0.00,0.40)	0.20 (0.10,0.30)	− 0.546	0.585
	Standing^#^	0.20 (0.00,0.25)	0.10 (0.00,0.40)	− 0.583	0.560

^#^Non-parametric tests were employed to analyze data that did not follow a normal distribution.

### Spatiotemporal Parameters

During stair ascent, the scoliosis group displayed a slower walking speed (scoliosis: 0.55; control: 0.58 m/s) and narrower step width (scoliosis: 0.09; control: 0.11 m) compared to the control group (*P* < 0.05). Moreover, the scoliosis group exhibited longer support time on both the concave side (scoliosis: 0.82; control: 0.77 s) and the convex side (scoliosis: 0.83; control: 0.77 s) in comparison to the control group (*P* < 0.05). Regarding stair descent, the scoliosis group had a narrower step width (scoliosis: 0.12; control: 0.14 m) than the control group (*P* < 0.05). Additionally, the scoliosis group demonstrated longer support time on the convex side (scoliosis: 0.75; control: 0.70 s) compared to the control group (*P* < 0.05). However, there were no statistically significant differences found in other spatiotemporal parameters (*P* > 0.05).

### Kinematics

During the stair ascending activity, the scoliosis group exhibited a slower walking speed compared to the control group (scoliosis: 0.55; control: 0.58 m/s). The difference between the two groups was analyzed using covariance analysis. The results presented in Table 2 indicate that the scoliosis group displayed greater hip joint adduction and abduction on the concave side (scoliosis: 16.69°; control: 15.77°), as well as greater hip joint internal and external rotation on the convex side (scoliosis: 12.87°; control: 10.90°). Moreover, the scoliosis group showed a higher bilateral asymmetry index for hip joint internal and external rotation (scoliosis: 16.31; control: 0.85) and a more pronounced asymmetry index for hip joint adduction and abduction on both sides (scoliosis: − 5.27; control: 2.64), compared to the control group (*P* < 0.05). Additionally, the scoliosis group exhibited lower hip joint flexion and extension on the concave side (scoliosis: 60.38°; control: 62.07°) and lower knee joint flexion and extension on the concave side (scoliosis: 89.75°; control: 92.41°) as compared to the control group (*P* < 0.05).

**Table 2:** Kinematics between group analysis (ascent) [$\overline{x}$ ± *s*, M(P25, P75)].

Joint	Direction		Concave/Convex	Scoliosis (n = 28)	Control (n = 28)	t/Z	*P* value
H ip	F/E	ROM (°)	Convex^#^	61.70 (58.08,65.66)	61.95 (59.48,65.98)	− 0.812	0.417
			**Concave**^#^	**60.38 (57.35,62.96)**	**62.07 (59.68,65.39)**	**− 3.816**	< 0.001***
		AI (%)^#^		1.61 (− 4.83,8.87)	0.66 (− 5.29,6.02)	− 1.552	0.121
	Add/Abd	ROM (°)	Convex^#^	16.21 (12.96,19.02)	16.56 (12.82,19.22)	− 0.401	0.688
			**Concave**	**16.69 ± 4.20**	**15.77 ± 4.12**	**− 1.976**	0.049*
		**AI** (**%**)		**− 5.27 ± 26.59**	**2.64 ± 17.81**	**3.148**	0.002**
	IR/ER	ROM (°)	**Convex**^#^	**12.87 (9.84,15.29)**	**10.90 (9.28,13.09)**	**− 4.267**	< 0.001***
			Concave^#^	10.52 (8.75,12.62)	10.91 (9.53,12.83)	− 1.181	0.237
		**AI** (**%**)^**#**^		**16.31 ± 33.51**	**0.85 ± 28.79**	**− 4.400**	< 0.001***
K n ee	F/E	ROM (°)	Convex	91.84 ± 7.33	92.63 ± 7.87	0.919	0.359
			**Concave**^#^	**89.75 (86.45,94.58)**	**92.41 (88.09,97.02)**	**− 2.966**	0.033*
		AI (%)^#^		101.24 (96.41,105.04)	99.79 (94.59,105.08)	− 1.721	0.085
Ankle	DF/PF	ROM (°)	Convex^#^	45.00 (41.68,49.24)	47.28 (41.07,52.11)	− 1.864	0.062
			Concave^#^	45.59 (40.40,52.45)	46.59 (42.97,51.16)	− 1.394	0.163
		AI (%)^#^		− 2.82 (− 12.28,9.94)	− 0.22 (− 9.17,5.41)	− 0.3345	0.730

*AI* asymmetry index, *F* flexion, *E* extension, *Add* adduction, *Abd* abduction, *IR* internal rotation, *ER* external rotation, *DF* dorsiflexion, *PF* plantarflexion.

^#^Non-parametric tests were employed to analyze data that did not follow a normal distribution.

*Compared to the control group, *P* < 0.05.

**Compared to the control group, *P* < 0.01.

***Compared to the control group, *P* < 0.001.

According to the results presented in Table 3, the scoliosis group exhibited higher motion of hip joint adduction/abduction (scoliosis: 12.38°; control: 10.75°), asymmetrical index of hip joint adduction/abduction (scoliosis: 10.46; control: − 2.68), and hip joint rotation on the convex side (scoliosis: 16.05°; control: 12.44°) during the descent when compared to the control group (*P* < 0.05).

**Table 3:** Kinematics between group analysis (descent) [$\overline{x}$ ± *s*, M(P25, P75)].

Joint	Direction		Concave/Convex	Scoliosis (n = 28)	Control (n = 28)	t/Z	*P* value
Hip	F/E	ROM (°)	Convex^#^	28.51 (25.27,32.55)	28.21 (25.97,32.00)	− 0.420	0.674
			Concave^#^	29.65 (26.99,33.33)	30.31 (27.28,34.23)	− 0.168	0.866
		AI (%)^#^		− 4.82 (− 18.06,9.93)	− 4.01 (− 18.22,6.53)	− 0.416	0.678
	Add/Abd	ROM (°)	**Convex**^#^	**12.38 (10.22,14.48)**	**10.75 (7.95,14.23)**	**− 2.279**	0.023*
			Concave	11.37 (8.77,13.17)	11.14 (9.02,13.74)	− 0.014	0.989
		**AI** (**%**)		**10.46 (− 7.19,31.06)**	**− 2.68 (− 16.73,15.00)**	**− 2.611**	0.009**
	IR/ER	ROM (°)	**Convex**^#^	**16.05 (13.21,18.93)**	**12.44 (10.36,15.90)**	**− 4.100**	< 0.001***
			Concave^#^	15.24 ± 2.98	14.35 ± 3.84	− 1.490	0.139
		AI (%)^**#**^		5.10 (− 19.19,27.92)	− 5.57 (− 26.87,18.57)	− 1.868	0.062
Knee	F/E	ROM (°)	Convex	96.76 ± 5.38	98.00 ± 7.60	1.056	0.293
			Concave^#^	95.32 ± 5.40	97.39 ± 7.21	1.828	0.070
		AI (%)^#^		1.50 ± 6.01	0.59 ± 7.76	− 0.732	0.466
Ankle	DF/PF	ROM (°)	Convex^#^	66.70 ± 5.66	66.17 ± 6.29	− 0.501	0.617
			Concave^#^	67.43 ± 5.04	66.06 ± 5.47	− 1.489	0.139
		AI (%)^#^		− 1.77 (− 4.80,3.05)	− 0.73 (− 5.62,4.55)	− 0.626	0.531

^#^Non-parametric tests were employed to analyze data that did not follow a normal distribution.

*Compared to the control group, *P* < 0.05.

**Compared to the control group, *P* < 0.01.

***Compared to the control group, *P* < 0.001.

### Spinal horizontal plane relative motion

Covariance analysis was conducted to account for the influence of walking speed on the comparison between the scoliosis group and the control group during stair ascending activity, as the scoliosis group exhibited a lower walking speed compared to the control group. The results in Table 4 indicate that during stair ascent, the scoliosis group demonstrated significantly higher values (*P* < 0.05) than the control group for the maximum shoulder-pelvis of convexity (scoliosis: 9.61°; control: 7.17°), bilateral asymmetry index of shoulder-pelvis (scoliosis: 66.04; control: 8.33), maximum shoulder-trunk of concavity (scoliosis: − 21.61; control: − 45.14°), maximum thorax-pelvis of concavity (scoliosis: 2.85°; control: 1.98°), and bilateral asymmetry index of thorax-pelvis (scoliosis: − 88.28; control: − 71.57). Conversely, the scoliosis group exhibited significantly lower values (*P* < 0.05) than the control group for the maximum shoulder-pelvis of concavity (scoliosis: 4.82°; control: 6.97°), maximum shoulder-trunk of convexity (scoliosis: 3.91°; control: 7.09°), and maximum thorax-pelvis of convexity (scoliosis: 0.96°; control: 1.21°).

**Table 4:** Spinal relative motion in horizontal plane between group analysis (ascent)[$\overline{x}$ ± *s*, M(P25, P75)].

Segment		Scoliosis (n = 28)	Control (n = 28)	t/Z	*P* Value
**Shoulder-Pelvis**	**Max of convexity**	**9.61 ± 4.97**	**7.17 ± 3.62**	**− 5.015**	< 0.001***
	**Max of concavity**	**4.82 (1.54,8.97)**	**6.97 (4.74,9.51)**	**− 4.307**	< 0.001***
	**AI** (**%**)^**#**^	**66.04 (− 23.58,146.25)**	**8.33 (− 43.96,46.18)**	**− 4.714**	< 0.001***
Shoulder-Trunk	**Max of convexity**	**3.91 (− 0.99,7.89)**	**7.09 (5.32,9.51)**	**− 5.835**	< 0.001***
	**Max of concavity**	**− 21.61 ± 6.63**	**− 45.14 ± 8.39**	**− 2.654**	0.008**
	AI (%)^#^	− 25.39 (− 218.50,45.59)	− 8.13 (− 212.06,9.86)	− 0.110	0.912
**Thoracic-Pelvis**	**Max of convexity**^#^	**0.96 (− 13.76,1.64)**	**1.21 (0.11,2.42)**	**− 2.465**	0.014*
	**Max of concavity**^#^	**2.85 (1.85,4.06)**	**1.98 (0.65,2.80)**	**− 5.982**	< 0.001***
	**AI** (**%**)^**#**^	**− 88.28 (− 213.30,− 12.73)**	**− 71.57 (− 182.75,118.87)**	**− 2.352**	0.019*

*AI* asymmetry index, *Max* maximum.

^#^Non-parametric tests were employed to analyze data that did not follow a normal distribution.

*Compared to the control group, *P* < 0.05.

**Compared to the control group, *P* < 0.01.

***Compared to the control group, *P* < 0.001.

The results from Table 5 indicate that, during the descent of stairs, the scoliosis group exhibited higher maximum values for shoulder-pelvic of convexity (scoliosis: 6.28°; control: 2.61°), shoulder-pelvic of concavity (scoliosis: 6.87°; control: 4.24°), and shoulder-trunk maximum asymmetry index (scoliosis: − 9.99; control: 3.80) compared to the control group (*P* < 0.05). Conversely, the scoliosis group had lower maximum values for shoulder-trunk of convexity (scoliosis: 4.93; control: 7.11°) and shoulder-trunk of concavity (scoliosis: 3.98°; control: 6.86°) when compared to the control group (*P* < 0.05).

**Table 5:** Spinal relative motion in horizontal plane between group analysis (descent) [$\overline{x}$ ± *s*, M(P25, P75)].

Segment		Scoliosis (n = 28)	Control (n = 28)	t/Z	*P* Value
**Shoulder-Pelvis**	**Max of convexity**	**6.28 (3.31,8.75)**	**2.61 (1.14,5.51)**	**− 4.378**	< 0.001***
	**Max of concavity**	**6.87 ± 4.12**	**4.24 ± 2.98**	**− 4.141**	< 0.001***
	AI (%)^#^	0.12 (− 12.11,7.48)	− 18.63 (− 54.76,33.93)	− 1.294	0.196
Shoulder-Trunk	**Max of convexity**	**4.93 ± 4.16**	**7.11 ± 3.74**	**3.123**	0.002**
	**Max of concavity**	**3.98 ± 4.30**	**6.86 ± 3.32**	**4.184**	< 0.001***
	**AI** (**%**)^**#**^	**− 9.99 (− 34.78,− 2.26)**	**3.80 (− 4.42,25.01)**	**− 3.002**	0.003**
**Thoracic-Pelvis**	Max of convexity^#^	1.43 (0.44,3.00)	1.38 (0.56,2.71)	− 0.069	0.945
	Max of concavity^#^	1.87 ± 1.86	2.41 ± 2.77	1.286	0.201
	AI (%)^#^	− 10.79 ± 0.46	0.20 ± 0.71	0.599	0.553

^#^Non-parametric tests were employed to analyze data that did not follow a normal distribution.

*Compared to the control group, *P* < 0.05.

**Compared to the control group, *P* < 0.01.

***Compared to the control group, *P* < 0.001.

### Kinetics

To analyze the differences between the two groups, covariance analysis was employed to correct for the influence of ascending speed. The findings presented in Table 6 revealed that during stair ascent, the scoliosis group demonstrated a greater asymmetry index in center of mass displacement (scoliosis: 0.68; control: − 0.50), knee joint convex side peak torque (scoliosis: 1.38; control: 1.26 NM/Kg), knee joint concave side peak torque (scoliosis: 1.38; control: 1.25 NM/Kg), and asymmetry index in knee joint peak torque (scoliosis: 4.25; control: − 0.44) (*P* < 0.05). Conversely, when considering concave side center of mass displacement (scoliosis: 0.26; control: 0.26 m), hip joint convex side peak torque (scoliosis: 0.52; control: 0.66 NM/Kg), hip joint concave side peak torque (scoliosis: 0.54; control: 0.63 NM/Kg), ankle joint concave side peak torque (scoliosis: 1.40; control: 1.49 N*M/Kg), asymmetry index in ankle joint peak torque (scoliosis: − 0.28; control: − 2.63), peak value of ground reaction force in the medial–lateral direction (scoliosis: 0.06; control: 0.06 N/Kg), peak value of ground reaction force in the vertical direction on the convex side (scoliosis: 1.19; control: 1.22 N/Kg), and peak value of ground reaction force in the vertical direction on the concave side (scoliosis: 1.17; control: 1.23 N/Kg), the scoliosis group exhibited lower values than the control group (*P* < 0.05).

**Table 6:** Kinetics between group analysis (ascent) [$\overline{x}$ ± *s*, M(P25, P75)].

		Scoliosis (n = 28)	Control (n = 28)	t/Z	*P* value
Center of gravity (m)	Displacement of convex	0.26 ± 0.01	0.26 ± 0.01	− 0.113	0.910
	**Displacement of concave**^#^	**0.26 (0.25,0.26)**	**0.26 (0.25,0.27)**	**− 3.673**	< 0.001***
	**AI** (**%**)^**#**^	**0.68 (− 3.24,4.88)**	**− 0.50 (− 4.37,2.96)**	**− 2.104**	0.035*
Joint peak moment flexion–extension (N*M/Kg)	**Hip-convexity**^**#**^	**0.52 (0.43,0.62)**	**0.66 (0.50,0.75)**	**− 5.055**	< 0.001***
	**Hip-concavity**^**#**^	**0.54 (0.48,0.66)**	**0.63 (0.50,0.74)**	**− 3.369**	0.001**
	AI (%)^#^	− 7.65 (− 24.62,16.20)	− 1.16 (− 19.79,21.52)	− 1.569	0.117
	**Knee-convexity**^**#**^	**1.38 (1.25,1.60)**	**1.26 (1.10,1.38)**	**− 5.743**	< 0.001***
	**Knee-concavity**^**#**^	**1.38 (1.21,1.51)**	**1.25 (1.08,1.43)**	**− 3.587**	< 0.001***
	**AI** (**%**)^**#**^	**4.25 (− 5.92,14.01)**	**− 0.44 (− 10.87,9.94)**	**− 2.251**	0.024*
	Ankle-convexity^#^	1.41 (1.27,1.53)	1.45 (1.33,1.53)	− 1.659	0.097
	**Ankle-concavity**^**#**^	**1.40 (1.31,1.49)**	**1.49 (1.39,1.56)**	**− 4.513**	< 0.001***
	**AI** (**%**)^**#**^	**− 0.28 (− 6.15,5.82)**	**− 2.63 (− 8.57,2.44)**	**− 2.124**	0.034*
Ground reaction force (N/Kg)	AP convexity^#^	7.56 (6.48,8.81)	0.08 (0.07,0.09)	− 1.027	0.305
	AP concavity^#^	7.77 (6.39,8.86)	0.07 (0.06,0.09)	− 0.646	0.518
	AI (%)^#^	− 0.25 (− 16.63,12.97)	2.75 (− 9.71,17.06)	− 1.692	0.091
	ML convexity^#^	0.06 (0.06,0.08)	0.06 (0.05,0.08)	− 1.287	0.198
	ML concavity^#^	0.06 (0.05,0.07)	0.06 (0.05,0.10)	− 2.596	0.009
	AI (%)^#^	6.51 (− 16.80,25.85)	3.40 (− 18.72,20.49)	− 1.607	0.108
	**V convexity**^#^	**1.19 (1.11,1.24)**	**1.22 (1.17,1.29)**	**− 4.386**	< 0.001***
	**V concavity**^#^	**1.17 (1.11,1.24)**	**1.23 (1.16,1.29)**	**− 4.382**	< 0.001***
	AI (%)^#^	0.21 (− 4.22,4.68)	0.21 (− 0.28,3.99)	− 0.149	0.882

Peak joint moment: It refers the maximum moment value experienced by individual lower limb joints during flexion and extension movements. It serves as a primary indicator of the loading conditions in the sagittal plane for these joints.

*AI* Asymmetry Index, *AP* Anterior–Posterior, *ML* Medial–Lateral, *V* Vertical.

^#^Non-parametric tests were employed to analyze data that did not follow a normal distribution.

*Compared to the control group, *P* < 0.05.

**Compared to the control group, *P* < 0.01.

***Compared to the control group, *P* < 0.001.

The results presented in Table 7 indicate that, during the descent stairs, the scoliosis group exhibited a higher peak knee joint moment (scoliosis: 1.17; control: 1.08 NM/Kg) and vertical ground reaction force in the lateral-medial direction (scoliosis: 0.10; control: 0.09 N/Kg) compared to the control group (*P* < 0.05). Additionally, the scoliosis group displayed a lower peak hip joint moment (scoliosis: 0.12; control: 0.16 NM/Kg), vertical ground reaction force in the anterior–posterior direction (scoliosis: 0.14; control: 0.15 N/Kg), vertical ground reaction force in the lateral-medial direction (scoliosis: 0.10; control: 0.11 N/Kg), and bilateral asymmetry index for vertical ground reaction force in the lateral-medial direction (scoliosis: −3.26; control: − 4.79) compared to the control group (*P* < 0.05).

**Table 7:** Kinetics between group analysis (descent) [$\overline{x} \pm s$, M(P25, P75)].

		IS组(n = 28)	对照组 (n = 28)	t/Z值	*P*值
Center of gravity (m)	**Displacement of convex**	**0.28 ± 0.02**	**0.28 ± 0.02**	**− 2.186**	0.030*
	Displacement of concave^#^	0.28 ± 0.02	0.27 ± 0.01	− 1.812	0.071
	AI (%)^#^	0.36 ± 0.07	0.08 ± 0.07	− 0.282	0.779
Joint peak moment flexion–extension (N*M/Kg)	**Hip-convexity**^**#**^	**0.12 (0.06,0.20)**	**0.16 (0.07,0.26)**	**− 1.995**	0.046*
	Hip-concavity^#^	0.13 (0.05,0.24)	0.16 (0.06,0.27)	− 1.476	0.140
	AI (%)^#^	− 15.89 (− 102.79,63.44)	− 1.26 (− 58.12,61.24)	− 1.222	0.222
	**Knee-convexity**^**#**^	**1.17 (1.04,1.34)**	**1.08 (0.95,1.32)**	**− 3.158**	0.002**
	Knee-concavity^#^	1.15 (1.02,1.28)	1.10 (0.90,1.32)	− 1.757	0.079
	AI (%)^#^	5.11 (− 7.53,16.23)	− 2.54 (− 12.11,11.75)	− 1.562	0.118
	Ankle-convexity^#^	1.33 (1.18,1.48)	1.34 (1.20,1.47)	− 0.030	0.976
	Ankle-concavity^#^	1.22 (1.11,1.41)	1.29 (1.14,1.47)	− 1.706	0.088
	AI (%)^#^	5.60 (− 5.42,16.24)	3.38 (− 8.60,11.88)	− 1.038	0.299
Ground reaction force (N/Kg)	**AP convexity**^#^	**0.14 (0.12,0.16)**	**0.15 (0.13,0.16)**	**− 2.646**	0.008**
	**AP concavity**^#^	0.14 (0.13,0.16)	0.15 (0.13,0.17)	− 1.445	0.148
	AI (%)^#^	0.94 (− 13.61,13.79)	− 1.19 (− 9.21,13.34)	− 0.127	0.899
	**ML convexity**^#^	**0.10 (0.08,0.12)**	**0.09 (0.06,0.11)**	**− 2.644**	0.008**
	**ML concavity**^#^	**0.10 (0.08,0.12)**	**0.11 (0.09,0.16)**	**− 2.191**	0.028*
	**AI** (**%**)^**#**^	**− 3.26 (− 17.11,14.12)**	**− 4.79 (− 52.61,13.83)**	**− 1.697**	0.090*
	V convexity^#^	1.43 (1.35,1.60)	1.49 (1.40,1.58)	− 1.290	0.197
	V concavity^#^	1.43 (1.33,1.60)	1.47 (1.35,1.59)	− 0.883	0.377
	AI (%)^#^	0.88 ± 0.08	2.60 ± 0.08	1.446	0.150

^#^Non-parametric tests were employed to analyze data that did not follow a normal distribution.

*Compared to the control group, *P* < 0.05.

**Compared to the control group, *P* < 0.01.

***Compared to the control group, *P* < 0.001.

## Discussion

The findings of the current study indicate that patients with scoliosis exhibit reduced range of motion in the relative rotation of the shoulder-pelvis, shoulder -trunk, and thorax-pelvis during stair ascent when compared to the control group (*P* < 0.05). These results suggest that individuals with scoliosis have limited flexibility in the aforementioned rotations, which reflects a more rigid movement pattern. The activity of ascending stairs requires enhanced trunk control in comparison to walking on level ground. It poses a greater challenge for maintaining balance and necessitates heightened coordination among the joints. The study uncovered stiffness in the rotational coordination among the shoulder girdle, thoracic and pelvis, resulting in patients limiting movement on the horizontal plane to maintain spinal stability. Previous research has shown that scoliosis is associated with increased stiffness and reduced flexibility in movement due to alterations in the three-dimensional structure of the spine^22^. In order to restrict the progression of coronal plane deformity, individuals with scoliosis minimize dynamic movement of their trunk. To primarily limit the progression of coronal plane deformity, individuals with scoliosis reduce dynamic movement of their trunk^23^. This consistent with the findings of our study, spinal stability is maintained and the progression of lateral curvature is further mitigated through enhanced stability in the sagittal and horizontal planes, achieved by reducing movement during dynamic activities.

The results of the analysis on lower limb kinematics and kinetics suggest that individuals with scoliosis exhibit decreased walking speed, step width, sagittal plane COM displacement on the concave side, peak AP GRF, peak sagittal plane hip joint moments, and peak sagittal plane ankle joint moments when compared to the control group during ascending stairs activity (*P* < 0.05). Conversely, these individuals demonstrate increased support time, sagittal plane hip joint flexion–extension range of motion on the concave side, sagittal plane hip joint rotation range of motion on the convex side, sagittal plane knee joint flexion–extension range of motion on the concave side, and peak sagittal plane knee joint moments (*P* < 0.05). The findings indicate that individuals with scoliosis experience an elongated support phase on both sides, improved flexion–extension and rotational flexibility in the hip joint on the concave and convex sides, heightened flexion–extension flexibility in the knee joint on the concave side, and increased load on both knee joints. In walking, the vertical displacement of the center of mass is crucial, particularly in the sagittal plane during stair ascending due to the combined upward and forward motion involved. The movement of the pelvis and hip joint in the sagittal plane is crucial for increasing the vertical displacement of the COM. This plays a significant role in optimizing work efficiency and minimizing energy expenditure during walking^24^. The anterior tilt of the body enhances the hip joint's range of motion in flexion and extension, consequently shifting the COM forward^25^. Our study discovered an augmented range of motion in flexion–extension specifically in the hip and knee joints of scoliosis patients. We propose that this phenomenon is possibly attributed to the spinal deformity of scoliosis patients, resulting in a shift in body weight toward the concave side. Consequently, the concave side experiences an increased load, which leads to the observed increase in flexion–extension range of motion in the hip and knee joints. However, the displacement of the center of gravity in the sagittal plane on the concave side is smaller compared to the control group. This suggests that the compensatory load does not effectively facilitate stair ascent. In order to address this issue, individuals with scoliosis employ compensation strategies such as diminishing their walking speed and extending their bilateral support time, thereby ensuring adequate balance and stability during the ascent of stairs.

Furthermore, our findings revealed that scoliosis patients exhibited reduced peak AP GRF on both the concave and convex sides, as well as lower peak hip joint moments in comparison with the control group. These findings indicating a lack of adequate propulsive force during stair ascent. Consistent with prior research, scoliosis patients demonstrate decreased stability during the support phase^26^, resulting in a decrease in walking speed. This decline in speed could be attributed to the gradual adaptation of the diminished support phase control mechanism^27^. Collectively, these findings suggest that scoliosis patients experience inadequate propulsion in the lower limbs during stair activities, which may necessitate higher energy expenditure compared to the control group to maintain functional efficiency. Consequently, rehabilitation programs should emphasize the strengthening of the lower limb muscle groups.

In terms of gait symmetry during stair ascent activities, the findings indicate that scoliosis patients demonstrated significantly higher asymmetry compared to the control group in various measures. These include the index of bilateral center of gravity displacement in the sagittal plane, the index of bilateral knee joint sagittal plane moment peak asymmetry, the index of bilateral ankle joint sagittal plane moment peak asymmetry, the index of shoulder-pelvis relative rotation asymmetry, and the index of thorax-pelvis relative rotation asymmetry (*P* < 0.05). The findings indicate that scoliosis patients demonstrate consistent asymmetry between the shoulder, thorax and pelvis during stair ascent. The observed asymmetry is likely caused by the rotational deformity in the spine, which consequently results in irregular movement of the joints. Previous studies have also corroborated the presence of asymmetry in GRF across three directions in scoliosis patients^26,28,29^. Notably, this study observed significant GRF asymmetry in the knee and ankle joints of scoliosis patients in the sagittal plane, aligning with previous research outcomes. Considering that the upper body represents approximately two-thirds of the total body weight, it is plausible that alterations in GRF primarily stem from the spinal deformity^30^. Currently, there is a lack of research establishing whether scoliosis patients have a higher susceptibility to knee and ankle joint injuries compared to the healthy population. However, a study conducted on individuals with low back pain showed a significantly higher occurrence of both low back and knee pain during community walking on slopes and stairs, as opposed to walking on level ground^31^. Furthermore, our study suggests that scoliosis patients experience asymmetrical loading in the lower limbs when ascending stairs. This specific movement pattern has the potential to cause uneven stress on both legs, thereby increasing the likelihood of joint injury in the limb bearing the greater load over a prolonged period. Examining changes in lower limb load among scoliosis patients can play a crucial role in adopting an early preventive approach to mitigating lower limb joint injuries through rehabilitation.

During the descent activity, this study observed a significant increase in shoulder-pelvic relative rotation range among scoliosis patients compared to the control group (*P* < 0.05). These findings suggest that scoliosis patients exhibit heightened flexibility in shoulder-pelvic movement on the horizontal plane during descent. However, there is currently a lack of research in the existing literature investigating the activity patterns of scoliosis patients during descent. This study offers the initial analysis of the motion patterns of scoliosis patients during descent, highlighting a distinction from the observed patterns during ascent. This disparity may arise from the inherent instability of descent, which is relatively less stable compared to ascending stairs. As a result, scoliosis patients who lack sufficient muscle compensation may encounter a higher risk of falling incidents during descent^32^. This finding reinforces the idea of reduced stability among scoliosis patients during descent activity.

The results of this study on lower limb kinematics and kinetics reveal that scoliosis patients display increased asymmetry in the range of motion of hip joint abduction–adduction and in the asymmetry of ML GRF when compared to the control group (*P* < 0.05). Additionally, scoliosis patients exhibit higher peak GRF on the convex side and lower peak GRF on the concave side in comparison to the control group (*P* < 0.05). The findings suggest that individuals with scoliosis undergo a shift in their center of gravity towards the convex side when engaging in descending activities. Descending primarily involves eccentric contraction, with the concave side still carrying a greater load. Additionally, there is poorer symmetry in hip joint adduction and abduction, resulting in an increased GRF on the convex side. To maintain balance during descent, there is an increase in displacement on the coronal plane, which leads to an enlargement of the base area^33^. Previous studies have revealed that scoliosis patients exhibit asymmetrical segmental motion in both the coronal and horizontal planes, resulting in asymmetrical ground reaction forces, particularly in the ML direction, when compared to the control group^34,35^. This asymmetry can potentially be attributed to the poorer balance control ability demonstrated by scoliosis patients during movement^23^. It is worth noting that while ascending activities do not demonstrate this asymmetrical motion pattern, descending activities primarily exhibit it in the lower limbs. This observation could be attributed to the spinal instability in the three-dimensional plane, necessitating compensatory movement in the lower limbs and ultimately resulting in a more pronounced asymmetrical pattern.

### Limitations

Our study comprised a cohort of individuals aged 18–20 years with Cobb angles measuring less than 30°. It is important to note that the findings may not encompass all aspects of scoliosis. To mitigate this limitation, forthcoming research could investigate strategies including increasing the sample size, expanding the age range, and conducting a more extensive biomechanical analysis that takes into account the severity classification of scoliosis.

## Conclusion

Scoliosis patients exhibit distinct movement patterns compared to the healthy population when performing stair activities. These patterns involve varying degrees of changes in relative motion in the sagittal plane, as well as alterations in lower limb kinematics and kinetics. During stair ascent, scoliosis patients display a seemingly more rigid movement pattern, while descent is characterized by an unstable pattern, potentially influenced by deformities in the three-dimensional plane of the spine. Additionally, lateral curvature in the coronal plane further amplifies asymmetrical motion between trunk segments and increases asymmetry in the lower limbs. Consequently, future rehabilitation treatment plans should prioritize addressing joint and muscle injuries in the lower limbs of scoliosis patients.

### Supplementary Information

Supplementary Information.

## Data Availability

The datasets used and analyzed during the current study available from the corresponding author on reasonable request.
