# Supplementary material for: Biomechanical analysis of trunk and lower limbs during stair activity in patients with scoliosis
Source: Sci Rep. 2024 Jun 24;14:14541. doi: 10.1038/s41598-024-65665-2 (PMC11196649; doi:10.1038/s41598-024-65665-2)
Supplement: Supplementary file 1 — Supplementary Information. [file 41598_2024_65665_MOESM1_ESM.pdf]

We verified all the data in Table 6 and confirmed that the statistical method used is the independent samples T-test.

We first checked the normal distribution based on the independent sample T-test method. The indicator that complies with the normal distribution is COM Displacement of convex .

Independent Samples Test

		Levene's Test for Equality of Variances		t-test for Equality of Means						
									95% Confidence Interval of the Difference	
		F	Sig.	t	df	Sig. (2-tailed)	Mean Difference	Std. Error Difference	Lower	Upper
Convex Foot COM Shift	Equal variances assumed	10.551	.001	-.110	301	.912	-.00014	.00129	-.00268	.00240
	Equal variances not assumed			-.113	294.707	.910	-.00014	.00127	-.00263	[illegible]

The remaining outcomes in Table 6 do not follow a normal distribution and require non-parametric testing.

The statistical results are as follows and all conform to the independent sample test results.

Nonparametric Tests

Hypothesis Test Summary

	Null Hypothesis	Test	Sig.	Decision
1	The distribution of Concave Foot COM Shift is the same across categories of 分组.	Independent-Samples Mann-Whitney U Test	.000	Reject the null hypothesis.
2	The distribution of COM SHIFT AI is the same across categories of 分组.	Independent-Samples Mann-Whitney U Test	.045	Reject the null hypothesis.
3	The distribution of Convex Ankle Moment_Moment_X_Max is the same across categories of 分组.	Independent-Samples Mann-Whitney U Test	.097	Retain the null hypothesis.
4	The distribution of Concave Ankle Moment_Moment_X_Max is the same across categories of 分组.	Independent-Samples Mann-Whitney U Test	.000	Reject the null hypothesis.
5	The distribution of AK MOMENT AI is the same across categories of 分组.	Independent-Samples Mann-Whitney U Test	.034	Reject the null hypothesis.
6	The distribution of Convex Hip Moment_Moment_X_Max is the same across categories of 分组.	Independent-Samples Mann-Whitney U Test	.000	Reject the null hypothesis.
7	The distribution of Concave Hip Moment_Moment_X_Max is the same across categories of 分组.	Independent-Samples Mann-Whitney U Test	.001	Reject the null hypothesis.
		Independent[illegible]		

8	The distribution of HP MOMENT AI is the same across categories of 分组.	Independent-Samples Mann-Whitney U Test	.117	Retain the null hypothesis.
9	The distribution of Convex Knee Moment_Moment_X_Max is the same across categories of 分组.	Independent-Samples Mann-Whitney U Test	.000	Reject the null hypothesis.
10	The distribution of Concave Knee Moment_Moment_X_Max is the same across categories of 分组.	Independent-Samples Mann-Whitney U Test	.000	Reject the null hypothesis.
11	The distribution of KN MOMENT AI is the same across categories of 分组.	Independent-Samples Mann-Whitney U Test	.024	Reject the null hypothesis.
12	The distribution of Convex GRF_A-P Peak is the same across categories of 分组.	Independent-Samples Mann-Whitney U Test	.305	Retain the null hypothesis.
13	The distribution of Concave GRF_A-P Peak is the same across categories of 分组.	Independent-Samples Mann-Whitney U Test	.518	Retain the null hypothesis.
14	The distribution of GRF AP PEAK AI is the same across categories of 分组.	Independent-Samples Mann-Whitney U Test	.091	Retain the null hypothesis.
15	The distribution of Convex GRF_M-L Peak is the same across categories of 分组.	Independent-Samples Mann-Whitney U Test	.198	Retain the null hypothesis.
16	The distribution of Concave GRF_M-L Peak is the same across categories of 分组.	Independent-Samples Mann-Whitney U	.009	Reject the null hypothesis.

**17**	The distribution of GRF ML PEAK AI is the same across categories of 分组.	Independent-Samples Mann-Whitney U Test	.108	Retain the null hypothesis.
**18**	The distribution of Convex GRF_Vertical Peak is the same across categories of 分组.	Independent-Samples Mann-Whitney U Test	.000	Reject the null hypothesis.
**19**	The distribution of Concave GRF_Vertical Peak is the same across categories of 分组.	Independent-Samples Mann-Whitney U Test	.000	Reject the null hypothesis.
**20**	The distribution of GRF V PEAK AI is the same across categories of 分组.	Independent-Samples Mann-Whitney U Test	.882	Retain the null hypothesis.

Asymptotic significances are displayed. The significance level is .05.

Double-click
activate
